# Seroconversion after SARS‐CoV‐2 vaccination is protective against severe COVID‐19 disease in heart transplant recipients

**DOI:** 10.1002/iid3.1086

**Published:** 2023-11-16

**Authors:** Szilvia Kugler, Dorottya Katalin Vári, Dániel Sándor Veres, Ákos Király, Tímea Teszák, Nóra Parázs, Zoltán Tarjányi, Zsófia Drobni, Zsófia Szakál‐Tóth, Gyula Prinz, Pál Miheller, Béla Merkely, Balázs Sax

**Affiliations:** ^1^ Department of Cardiology, Heart and Vascular Center Semmelweis University Budapest Hungary; ^2^ Faculty of Medicine Semmelweis University Budapest Hungary; ^3^ Department of Biophysics and Radiation Biology Semmelweis University Budapest Hungary; ^4^ Department of Surgery, Transplantation and Gastroenterology Semmelweis University Budapest Hungary

**Keywords:** antibody, COVID‐19, heart transplantation, immunosuppression, SARS‐CoV‐2, seroconversion, vaccination

## Abstract

**Background:**

Heart transplant (HTX) recipients are prone to develop complications after severe acute respiratory syndrome coronavirus 2 (SARS‐CoV‐2) infection. Vaccination is often ineffective due to weaker immunogenicity. In this high‐volume single‐center study, we aimed to determine factors influencing seroconversion after vaccination and predictors of severe SARS‐CoV‐2 infection.

**Methods:**

Two hundred twenty‐nine HTX recipients were enrolled. Type of the first two vaccine doses included messenger RNA (mRNA), vector, and inactivated vaccines. We carried out analyses on seroconversion after the second and third doses of vaccination and on severity of infection. Antispike protein SARS‐CoV‐2 immunoglobulin G (IgG) was measured after the second and third vaccines and serostatus was defined. Effect of the first two vaccine doses was studied on patients who did not suffer SARS‐CoV‐2 infection before antibody measurement (*n* = 175). The effectivity of the third vaccine was evaluated among seronegative recipients after the second vaccine (*n* = 53). Predictors for severe infection defined as pneumonia, hospitalization or death were assessed in all patients who contracted SARS‐CoV‐2 infection (*n* = 92).

**Results:**

62% of the recipients became seropositive after the second vaccination. Longer time between HTX and vaccination (odds ratio [OR]: 2.35) and mRNA vaccine (OR: 4.83) were predictors of seroconversion. 58% of the nonresponsive patients became seropositive after receiving the third vaccine. Male sex increased the chance of IgG production after the third dose (OR: 5.65). Clinical course of SARS‐CoV‐2 infection was severe in 32%. Of all parameters assessed, only seropositivity before infection was proven to have a protective effect against severe infection (OR: 0.11).

**Conclusions:**

We found that longer time since HTX, mRNA vaccine type, and male sex promoted seroconversion after SARS‐CoV‐2 vaccination in HTX recipients. Seropositivity—but not the number of vaccine doses—seemed to be protective against severe SARS‐CoV‐2 infection. Screening of HTX patients for anti‐SARS‐COV‐2 antibodies may help to identify patients at risk for severe infection.

## Graphical abstract

We found that longer time since heart transplantation (HTX), messenger RNA vaccine type, and male sex promoted seroconversion after SARS‐CoV‐2 vaccination in HTX recipients. Seropositivity—but not the number of vaccine doses—seemed to be protective against severe SARS‐CoV‐2 infection. Screening of HTX patients for anti‐SARS‐COV‐2 antibodies may help to identify patients at risk for severe infection.

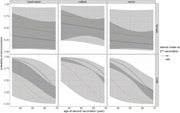

## 1 INTRODUCTION

Coronavirus disease 2019 (COVID‐19) is a pandemic affecting more than 600 million people worldwide and carrying a case fatality ratio of approximately 1% as of the beginning of December 2022.^1^ In Hungary, more than 2 million individuals (22% of the total population) contracted severe acute respiratory syndrome coronavirus 2 (SARS‐CoV‐2) in six pandemic waves with an overall case fatality ratio of 2.2%.^2^, ^3^, ^4^, ^5^, ^6^, ^7^ The Hungarian vaccination campaign was conducted with five different vaccines during the third wave of the COVID‐19 pandemic at the beginning of 2021. Two messenger RNA (mRNA) vaccines (BNT162b2—Pfizer‐BioNTech and mRNA‐1273—Moderna), two vector vaccines (AZD1222—AstraZeneca and Gam‐COVID‐Vac—Sputnik‐V), and one inactivated vaccine (HB02—Sinopharm) were equally widely used. All five vaccine types proved to be effective in the prevention of SARS‐CoV‐2 infection and COVID‐19‐related death during the third wave of the pandemic.^4^ Later, one more vector vaccine (Ad26.COV2.S—Janssen) became available. As of the middle of October 2022, more than 16 million vaccine doses have been administered in Hungary.^2^ During both Delta and Omicron waves, the risk of COVID‐19‐related death was lower in the primary immunized population compared to the unvaccinated individuals and the additional benefit of single and double booster vaccination against lethal outcome was also confirmed.^6^

Heart transplant (HTX) recipients suffering from SARS‐CoV‐2 infection were reported to have a case fatality ratio up to 28%.^8^ Vaccination has been proven to be effective in terms of fewer SARS‐CoV‐2 infections, hospitalizations, and deaths also among HTX recipients.^9^ While antibody response after a two‐dose vaccine schedule is relatively low among HTX recipients,^10^, ^11^, ^12^, ^13^ a third dose of vaccine markedly increase immunogenicity against SARS‐CoV‐2.^11^, ^12^ Based on these results, the European Society for Organ Transplantation (ESOT) recommends a third dose of SARS‐CoV‐2 vaccine for all solid organ transplant recipients.^14^

Predictors of severe and/or lethal SARS‐CoV‐2 infection in transplant recipients were reported to be unvaccinated status,^9^, ^15^ older age,^15^, ^16^ the use of a proliferation signal inhibitor, and the combination of calcineurin inhibitor, antimetabolite, and prednisone therapy.^16^

In this high‐volume single‐center prospective study, we examined the seroconversion rates achieved with various types and doses of SARS‐CoV‐2 vaccines and assessed factors influencing vaccine immunogenicity and predictors of disease severity in heart transplant recipients.

## 2 METHODS

### 2.1 Study cohort

The prospective cohort study of HTX recipients was conducted at the Heart and Vascular Center of Semmelweis University in Budapest, Hungary. We included all HTX patients between April 2020 and June 2022 who had either outpatient or inpatient visit at our clinic except 13 patients who had previous SARS‐CoV‐2 infection or immunization with SARS‐CoV‐2 vaccine before transplantation. A total of 229 patients were prospectively enrolled in the study over a period of 27 months. Database was closed at the end of June 2022. We carried out analyses on seroconversion after the second and third doses of SARS‐CoV‐2 vaccination and on severity of COVID‐19.

Demographic parameters, clinical data, details of immunosuppressive medication, dates of vaccinations, SARS‐CoV‐2 antibody levels, and information about COVID‐19 disease were collected from electronic health records. The study protocol has been approved by the Research Ethics Committee of the Medical Research Council of Hungary (No. IV/861‐1/2021/EKU). All patients signed informed consent.

### 2.2 Vaccination and antibody measurement

Vaccination status at the time of seroconversion or infection was defined as incomplete if the recipient had not received at least two vaccine doses or was <14 days after the second dose. Two‐, three‐, or four‐dose vaccination meant that at least 14 days passed after the respective vaccine dose.

Antispike (S) protein SARS‐CoV‐2 immunoglobulin G (IgG) levels were measured from blood plasma samples using the Elecsys Anti‐SARS‐CoV‐2S electrochemiluminescence immunoassay (Roche Diagnostics International Ltd.) detecting the receptor‐binding domain of spike protein. Greater than or equal to 0.8 U/mL was considered positive. Patients were declared to be seronegative if the serology was negative ≥14 days after the vaccination. Absolute values of IgG concentration were not included in the analyses of seroconversion because of the highly variable time period between the vaccination and antibody measurement.

### 2.3 Analysis of seroconversion

**Figure 1:**
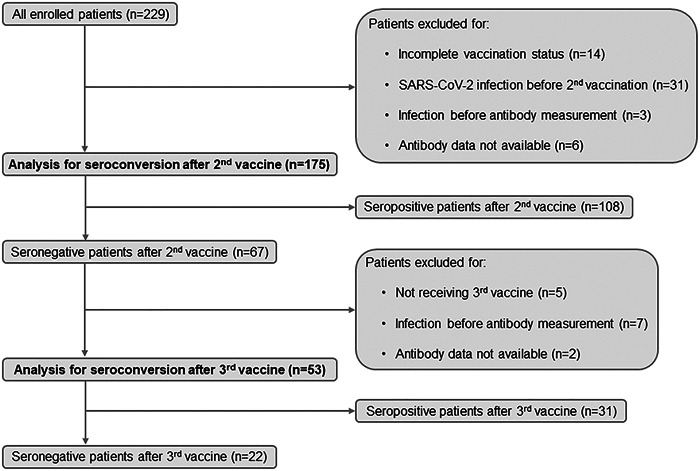
Flowchart of patient selection into the seroconversion analyses. Those recipients were enrolled into the analysis of the second vaccination who received complete vaccination and were not infected with severe acute respiratory syndrome coronavirus 2 (SARS‐CoV‐2) before antibody measurement. Effectivity of the third vaccination was investigated among those patients who did not reach seroconversion after the second vaccination. Recipients infected with SARS‐CoV‐2 before antibody measurement were excluded.

Figure 1 demonstrates the flowchart for patient selection into the seroconversion analyses. 94% of all patients received at least two‐dose SARS‐CoV‐2 vaccine during the study period (*n* = 215). Those who had SARS‐CoV‐2 infection before or <14 days after the second vaccine, those who suffered SARS‐CoV‐2 infection before antibody measurement and those who could not attend for antibody measurement were excluded from further analysis. One hundred seventy‐five patients were enrolled into the analysis for seroconversion after the second vaccine. The effect of the following parameters were evaluated: time between HTX and second vaccine, age and sex of the recipient, the immunosuppressive regime at the time of the second vaccination (steroid intake, mycophenolic acid [MPA], or mycophenolate mofetil (MMF) therapy, tacrolimus, and everolimus trough level) and type of the vaccine. Three patients who received different types of vaccines during their first and second vaccinations and five recipients who did not take tacrolimus were excluded from this analysis. Since only a few recipients took everolimus (*n* = 28), this parameter had to be removed from the calculations.

The second analysis of seroconversion was performed on seronegative patients after two doses (*n* = 67) who received a third vaccine. Patients who suffered SARS‐CoV‐2 infection after the third vaccine but before antibody measurement and those who could not attend for antibody measurement were excluded from the analysis. Consequently, this analysis included 53 recipients. During this examination, the effect of the following parameters were evaluated: time between HTX and third vaccine, age and sex of the recipient, steroid intake, MPA or MMF therapy, and tacrolimus trough level at the time of the third vaccination.

Patients who were excluded from seroconversion analyses could still be enrolled into the substudy of SARS‐SoV‐2 infection, if they contracted COVID‐19 during the study period.

### 2.4 Analysis of predictors for severe infection

From the 229 enrolled patients, everyone who contracted COVID‐19 during the study period was included in the analysis of predictors for severe infection regardless whether they were vaccinated and if yes, whether they contracted the infection before or after vaccination. In this subgroup of patients (*n* = 92), predictors for severe infection were assessed. Reinfection occurred in four patients was not included in analysis. Severe COVID‐19 was defined as at least one of the following criteria: radiologically proven pneumonia (either by chest X‐ray or by computed tomography scan), COVID‐19‐related hospitalization, or death due to SARS‐CoV‐2 infection. Relevant guideline of the National Institutes of Health differentiates mild, moderate, and severe COVID‐19. Individuals with mild illness do not have abnormal chest imaging, those with moderate illness may have chest imaging abnormality, while lung infiltrates >50% on chest imaging indicate severe illness.^17^ 2020 guidance of the World Health Organization also differentiates mild illness from pneumonias.^18^ In immunocompromised patients like heart transplant recipients, even moderate pneumonia may have severe consequences. As the exact extension of pulmonary infiltrates could not be judged in every case, we decided to differentiate mild infections from all others. Therefore, all patients with pneumonia were classified to the severe infection group. Whenever the patient required hospitalization, the infection was categorized as severe, since COVID‐19 as a systemic disease can lead to the insufficiency of other organs (e.g., renal failure) as well requiring in‐patient treatment.

In this analysis, the effect of the following parameters were evaluated on the severity: sex, age of the recipient, time between HTX and infection, vaccination before infection, serostatus before infection, time between the last vaccine and infection, steroid intake and MPA or MMF therapy at the onset of infection. Most important comorbidities as hypertension, diabetes mellitus, chronic obstructive pulmonary disease, and chronic kidney disease defined by glomerular filtration rate less than 30 mL/min/1.73 m^2^—were assessed for their additional possible confounding effects. In our sample, the presence of chronic kidney disease was very low, therefore we could not include it to the model.

### 2.5 Statistical analysis

Data were examined by descriptive statistics. Continuous variables are presented as mean (standard deviation) or median [Q_1_, Q_3_]. Categorical variables are presented as numbers (percentages).

Logistic regression model (with logit link) was used to analyze the relation between the seroconversion or severity of the infection (as an outcome variable) and the interested explanatory or possible confounder variables. Three independent models were fitted regarding the three different outcomes (seroconversion presence after the second and third vaccination and the severity of infection). At each model, two‐way interactions and nonlinear effect of explanatory variables was examined and included into the final model if it was relevant (based on descriptive statistics plots, clinical relevance, information criteria, and model diagnostics). The final model was found acceptable based on model diagnostics. Results are presented as odds ratios (ORs), 95% confidence intervals (CIs). *p* < 0.05 was considered statistically significant.

All statistical analyses were made with *R* statistical software^19^ using the table 1 package for descriptive tables^20^; the *ggplot2* package for descriptive plots^21^; the rms package for regression model calculations, and interpretations.^22^

## 3 RESULTS

### 3.1 Demography

Two hundred twenty‐nine adult patients were enrolled in this study. 76% of the recipients were male (*n* = 175). 94% of the patients (*n* = 215) received complete vaccination. Age of the patients varied between 21 and 79 years at the time of the second vaccination. Median age at the time of the second vaccination was 57 [48, 64] years for all recipients, 58 [50, 64] years for males, and 56 [44, 64] years for females. Median time between HTX and the second vaccination was 47 [23, 68] months. At the time of the second vaccination, 13% of the recipients (*n* = 29) were on steroid (methylprednisolone: *n* = 26, prednisolone: *n* = 3) therapy, 82% on MMF‐based (*n* = 121) or MPA‐based (*n* = 55) immunosuppression regime, 97% on tacrolimus (*n* = 209), 18% on everolimus (*n* = 38), and 1% received cyclosporine (*n* = 3).

### 3.2 Seroconversion after the second vaccine

In this cohort of patients, type of vaccination was mRNA vaccine in 68% (*n* = 117–105 Pfizer and 12 Moderna), vector vaccine in 26% (*n* = 45–44 AstraZeneca and 1 Janssen), and inactivated vaccine (Sinopharm) in 6% (*n* = 10).

62% of the recipients became seropositive after the second vaccine. Seropositive patients were significantly younger and had longer time since HTX. Two‐third of male patients but only half of the females reached seroconversion. Seroconversion rate was the highest (64%) among patients receiving mRNA vaccine. Recipients on steroids at the time of second vaccination were less likely to become seropositive (Table 1).

**Table 1:** Patient characteristics in the analysis of seroconversion after two doses of SARS‐CoV‐2 vaccine.

	Seronegative (*n* = 67)	Seropositive (*n* = 108)
Time between HTX and second vaccine (month)		
Mean (SD)	42.6 (41.5)	58.5 (36.4)
Median [Q1, Q3]	28.2 [16.4, 61.4]	55.9 [38.2, 71.0]
Age at second vaccination (year)		
Mean (SD)	56.5 (11.4)	54.9 (11.3)
Median [Q1, Q3]	58.9 [51.7, 64.9]	56.3 [48.3, 63.5]
Sex		
Female	21 (52.5%)	19 (47.5%)
Male	46 (34.1%)	89 (65.9%)
Type of first and second vaccine		
Inactivated	6 (60.0%)	4 (40.0%)
mRNA	42 (35.9%)	75 (64.1%)
Vector	18 (40.0%)	27 (60.0%)
Missing data	1 (33.3%)	2 (66.7%)
Steroid intake at second vaccination		
No	52 (33.5%)	103 (66.5%)
Yes	15 (75.0%)	5 (25.0%)
MMF/MPA therapy at second vaccination		
No	9 (31.0%)	20 (69.0%)
Yes	58 (39.7%)	88 (60.3%)
Tacrolimus trough level at second vaccination (ng/mL)		
Mean (SD)	7.77 (2.88)	6.41 (1.72)
Median [Q1, Q3]	6.84 [5.48, 9.47]	6.30 [5.29, 7.12]
Missing data	1 (20.0%)	4 (80.0%)
Everolimus trough level at second vaccination (ng/mL)		
Mean (SD)	3.84 (0.840)	3.39 (1.48)
Median [Q1, Q3]	4.00 [3.55, 4.15]	3.15 [2.63, 3.93]
Missing data	59 (40.1%)	88 (59.9%)

*Note*: Differences in demographic variables, type of vaccination, and immunosuppressive regime between seronegative and seropositive heart transplant recipients after two doses of SARS‐CoV‐2 vaccine.

Abbreviations: HTX, heart transplantation; MMF, mycophenolate mofetil; MPA, mycophenolic acid; mRNA, messenger RNA; Q1, first quartile; Q3, third quartile; SARS‐CoV‐2, severe acute respiratory syndrome coronavirus 2; SD, standard deviation.

**Figure 2:**
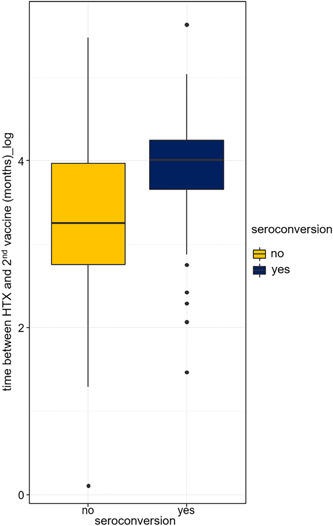
Time between heart transplantation and second vaccination on natural logarithmic scale. Time since heart transplantation was significantly higher in recipients who achieved seroconversion compared to the seronegative group.

**Figure 3:**
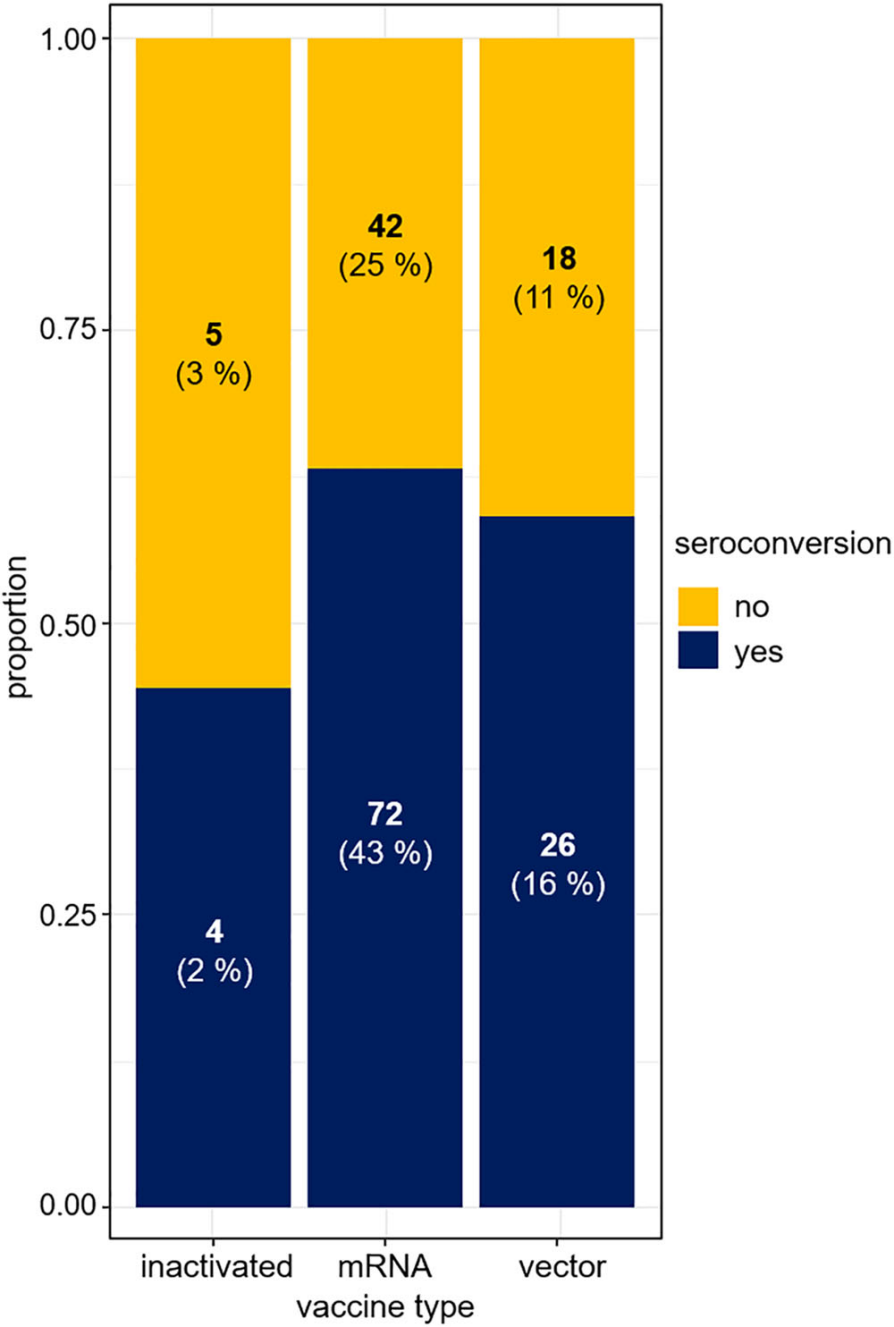
Proportion of vaccine types and the rate of seroconversion after the second vaccination in each group. Percentage of seropositivity was the highest among recipients receiving the messenger RNA (mRNA) type of vaccine and the lowest among patients getting the inactivated vaccine.

**Figure 4:**
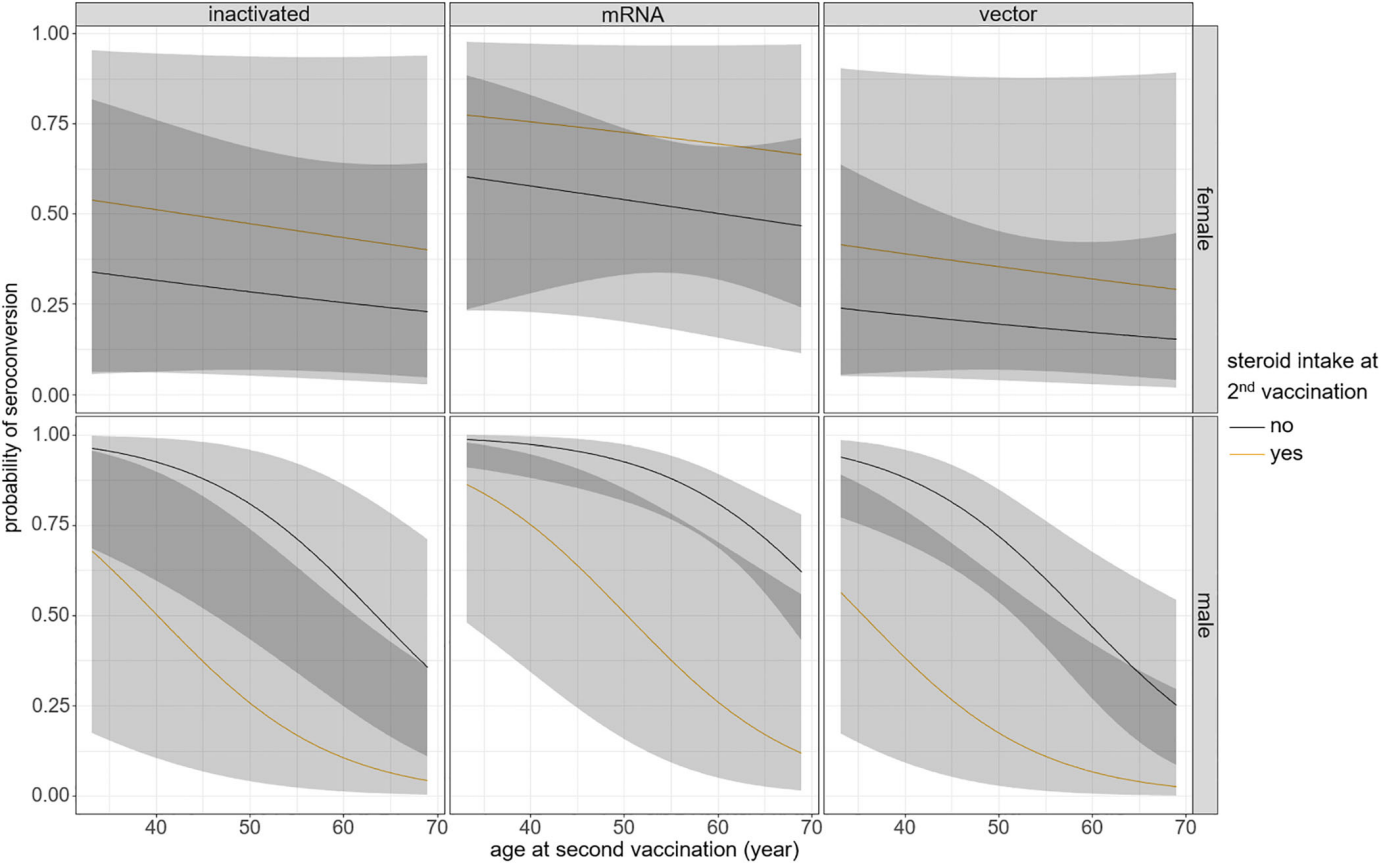
Effect of the significant variables on seroconversion after the second vaccination. Steroid intake decreased the probability of seroconversion among male recipients. In the female group, the effect of steroid is hard to interpret due to the small number of patients. For males, the chance of seroconversion decreased faster with age compared to females.

Significant predictors of seroconversion after second vaccination proved to be longer time between HTX and the second vaccination (OR: 2.35, 95% CI: 1.26–4.39, *p* = .007; Figure 2, Table 2) and vaccine type favoring mRNA vaccines (OR: 4.83, 95% CI: 1.33–17.5, *p* = .012; Figure 3, Table 2). Additionally, sex–age effect proved to be significant predictor in interaction for seroconversion. For male patients, the probability of seroconversion decreased faster with age compared to females. Seroconversion rate among patients on steroid therapy also depended on their sex: the seroconversion rate among male patients taking steroids (*n* = 16) was only 19%, while among female patients on steroid therapy (*n* = 4), this ratio was 50%. Consequently, for males, the steroid reduced the probability of seroconversion. For females, it seemed as if steroid intake somewhat increased the chance of seroconversion, but due to the small number of patients in this group, there was a high chance that this effect was caused by interindividual differences (Figures 4, Table 2).

**Table 2:** Predictors of seroconversion after two doses of SARS‐CoV‐2 vaccine.

Predictors	Seroconversion (yes vs. no seroconversion)		
	Odds ratios	95% CI	*p*
Time between HTX and second vaccine (month) (on logarithmic scale)	2.35	1.26–4.39	**.007**
Age at second vaccination (year)	0.98	0.93–1.04	.006
Sex (male vs. female)	1039.98	7.19–150395.28	.002
Type of first and second vaccine (inactivated vs. mRNA)	0.34	0.05–2.13	.3503^a^ (0.0104)
Type of first and second vaccine (inactivated vs. vector)	1.63	0.20–13.02	.8448^a^ (0.0104)
Type of first and second vaccine (mRNA vs. vector)	4.83	1.33–17.49	**.0116** ^a^ **(0.0104)**
Steroid intake at second vaccination (yes vs. no)	2.26	0.18–28.46	.0327
MMF/MPA therapy at second vaccination (yes vs. no)	1.19	0.42–3.36	.7389
Tacrolimus trough level at second vaccination (ng/mL) (on logarithmic scale)	0.40	0.08–1.93	.2519
Age at second vaccination (year) × Sex (male vs. female)^b^	0.91	0.84–0.99	.0296
Sex (male vs. female) × Steroid intake at second vaccination (yes vs. no)^b^	0.04	0.00–0.77	.0333
(Intercept)	0.15	0.00–37.39	

*Note*: Logistic regression analysis of the relation between different variables and seroconversion after two doses of SARS‐CoV‐2 vaccine. Time between HTX and vaccination and type of the vaccines had significant effect on seroconversion (*p*‐values are marked bold). Additionally, sex‐age and sex‐steroid effects proved to be significant predictors in interaction for seroconversion. Odds ratios given as seroconversion versus no seroconversion.

Abbreviations: CI, confidence interval; HTX, heart transplantation; MMF, mycophenolate mofetil; MPA, mycophenolic acid; *p*, Wald type *p* values; SARS‐CoV‐2, severe acute respiratory syndrome coronavirus 2.

^a^ Tukey adjusted *p* values for pairwise comparison.

^b^ Interaction term.

### 3.3 Seroconversion after the third vaccine

According to our institutional protocol, all patients included in this substudy (*n* = 53) received mRNA vaccine except one who asked for a vector vaccine. 58% of the enrolled patients became seropositive after receiving the third vaccine. In a logistic regression analysis, we found that sex had a significant effect on the seroconversion (male vs. female OR: 5.65, 95% CI: 1.61–22.7, *p* = .009). No significant effect on seroconversion was found for other variables (Tables 3 and 4).

**Table 3:** Patient characteristics in the analysis of seroconversion after the third dose of SARS‐CoV‐2 vaccine.

	Seronegative (*n* = 22)	Seropositive (*n* = 31)
Time between HTX and third vaccine (month)		
Mean (SD)	50.8 (59.6)	42.2 (26.8)
Median [Q1, Q3]	29.0 [19.9, 52.1]	32.1 [23.9, 55.6]
Age at third vaccination (year)		
Mean (SD)	58.0 (11.4)	56.4 (11.1)
Median [Q1, Q3]	58.9 [53.2, 66.3]	59.3 [53.1, 64.2]
Sex		
Female	12 (63.2%)	7 (36.8%)
Male	10 (29.4%)	24 (70.6%)
Steroid intake at third vaccination		
No	19 (40.4%)	28 (59.6%)
Yes	3 (50.0%)	3 (50.0%)
MMF/MPA therapy at third vaccination		
No	1 (20.0%)	4 (80.0%)
Yes	21 (43.8%)	27 (56.2%)
Tacrolimus trough level at third vaccination (ng/mL)		
Mean (SD)	6.95 (1.88)	7.40 (2.38)
Median [Q1, Q3]	6.59 (5.71, 7.77)	6.83 (5.69, 8.52)

*Note*: Differences in demographic variables, type of vaccination, and immunosuppressive regime between seronegative and seropositive heart transplant recipients after a third dose of SARS‐CoV‐2 vaccination. All 53 patients were seronegative after the first two vaccine doses.

Abbreviations: HTX, heart transplantation; MMF, mycophenolate mofetil; MPA, mycophenolic acid; Q1, first quartile; Q3, third quartile; SARS‐CoV‐2, severe acute respiratory syndrome coronavirus 2; SD, standard deviation.

**Table 4:** Predictors of seroconversion after the third dose of SARS‐CoV‐2 vaccine.

Predictors	Seroconversion (yes vs. no seroconversion)		
	Odds ratios	95% CI	*p*
Time between HTX and third vaccine (month) (on logarithmic scale)	1.33	0.57–3.30	.520
Age at third vaccination (year)	0.97	0.91–1.02	.235
Sex (male vs. female)	5.65	1.61–22.70	**.009**
Steroid intake at third vaccination (yes vs. no)	0.37	0.05–2.56	.308
Tacrolimus trough level at third vaccination (ng/mL) (on logarithmic scale)	2.33	0.27–23.64	.447
(Intercept)	0.27	0.00–168.99	

*Note*: Logistic regression analysis of the relation between different variables and seroconversion after the third dose of SARS‐CoV‐2 vaccination. Sex of the recipient was found to be a significant predictor of seroconversion (*p*‐value is marked bold). Odds ratios given as seroconversion versus no seroconversion.

Abbreviations: CI, confidence interval; *p*, Wald type *p* values; HTX, heart transplantation; SARS‐CoV‐2, severe acute respiratory syndrome coronavirus 2.

### 3.4 Seroconversion after the fourth vaccine

Among patients who were seronegative after the third vaccine (*n* = 22), 13 recipients received the fourth SARS‐CoV‐2 vaccine. Three recipients were infected with SARS‐CoV‐2 before their fourth vaccination, therefore, the effect of the fourth vaccine could not be evaluated in their cases. Only 40% of the remaining 10 patients became seropositive after the fourth vaccine.

### 3.5 Infection severity

Forty percent of all included patients (*n* = 92) contracted COVID‐19. Clinical course of SARS‐CoV‐2 infection was severe in 29 patients (32%) including need for hospitalization (*n* = 25), pneumonia verified by chest imaging (*n* = 24), and lethal outcome (*n* = 6). Two HTX recipients (one unvaccinated and one incompletely vaccinated) died during the third pandemic wave caused by the Alpha variant and four patients (one of them received a single dose, one of them got two doses and two of them got three doses of vaccination) died during the fourth (Delta) wave. In the case of COVID‐19 disease, treatment strategy (discontinuation or dose reduction of MMF/MPA therapy, azithromycin, steroid therapy, convalescent plasma, favipiravir, remdesivir, monoclonal antibody therapies, oxygen supplementation, mechanical ventilation) was chosen based on the severity of the disease and the actually available treatment options.

Based on the logistic regression model, we found that seropositive status of the recipients before infection had a significant protective effect against severe SARS‐CoV‐2 infection (OR: 0.11, 95% CI: 0.01–0.63, *p* = .019). On the contrary, other included variables had no significant effect on infection severity (Tables 5 and 6).

**Table 5:** Clinical data of patients suffering from COVID‐19 disease.

	Mild (*n* = 63)	Severe (*n* = 29)
Sex		
Female	19 (73.1%)	7 (26.9%)
Male	44 (66.7%)	22 (33.3%)
Age at infection (year)		
Mean (SD)	53.4 (12.0)	56.8 (10.4)
Median [Q1, Q3]	56.3 [46.5, 62.7]	61.4 [51.7, 64.4]
Hypertension		
No	15 (83.3%)	3 (16.7%)
Yes	48 (64.9%)	26 (35.1%)
Diabetes mellitus		
No	40 (71.4%)	16 (28.6%)
Yes	23 (63.9%)	13 (36.1%)
Chronic obstructive pulmonary disease		
No	51 (70.8%)	21 (29.2%)
Yes	12 (60%)	8 (40%)
Time between HTX and infection (month)		
Mean (SD)	57.7 (44.7)	45.9 (40.1)
Median [Q1, Q3]	52.2 [25.6, 75.8]	35.4 [18.6, 60.7]
Missing data	1 (100%)	0 (0%)
Vaccination before infection		
No or incomplete	27 (62.8%)	16 (37.2%)
Two	8 (61.5%)	5 (38.5%)
Three or more	28 (77.8%)	8 (22.2%)
Serostatus before infection		
Seronegative	28 (60.9%)	18 (39.1%)
Seropositive	23 (85.2%)	4 (14.8%)
Missing data	12 (63.2%)	7 (36.8%)
Time between last vaccine and infection (day)		
Mean (SD)	111 (67.3)	130 (53.5)
Median [Q1, Q3]	109 [57.3, 148]	137 [84.0, 165]
Missing data	27 (62.8%)	16 (37.2%)
Steroid intake at the onset of infection		
No	52 (71.2%)	21 (28.8%)
Yes	11 (57.9%)	8 (42.1%)
MMF/MPA therapy at the onset of infection		
No	14 (73.7%)	5 (26.3%)
Yes	49 (67.1%)	24 (32.9%)

*Note*: Differences in demographic variables, vaccination, serostatus and immunosuppressive regime between heart transplant recipients suffering from mild and severe COVID‐19 disease. All examined variables were determined at the onset of or shortly before the infection.

Abbreviations: HTX, heart transplantation; MMF, mycophenolate mofetil; MPA, mycophenolic acid; Q1, first quartile; Q3, third quartile; SD, standard deviation.

**Table 6:** Predictors of COVID‐19 infection severity.

Predictors	Severity of SARS‐CoV‐2 infection (severe vs. mild infection)		
	Odds ratios	95% CI	*p*
Sex (male vs. female)	2.99	0.67–17.28	.179
Age at infection (year)	1.02	0.96–1.09	.441
Hypertension (yes vs. no)	2.14	0.43–12.84	.370
Diabetes mellitus (yes vs. no)	1.02	0.26–4.00	.974
Chronic obstructive pulmonary disease (yes vs. no)	0.98	0.18–4.79	.985
Time between HTX and infection (month) (on logarithmic scale)	0.60	0.27–1.21	.175
Vaccination before infection (yes vs. no)	3.60	0.66–23.33	.151
Serostatus before infection (seropositive vs. seronegative)	0.11	0.01–0.63	**.019**
Steroid intake at the onset of infection (yes vs. no)	1.55	0.24–8.98	.628
MMF/MPA therapy at the onset of infection (yes vs. no)	1.46	0.29–8.88	.658
(Intercept)	0.12	0.00–13.78	

*Note*: Logistic regression analysis of the relation between different variables and the severity of SARS‐CoV‐2 infection. Seropositive status had significant protective effect on the severity of SARS‐CoV‐2 infection (*p*‐value is marked bold). Odds ratios given as severe versus mild infection.

Abbreviations: CI, confidence interval; HTX, heart transplantation; MMF, mycophenolate mofetil; MPA, mycophenolic acid; *p*, Wald type *p* values; SARS‐CoV‐2, severe acute respiratory syndrome coronavirus 2.

## 4 DISCUSSION

SARS‐CoV‐2 infection has been associated with increased morbidity and mortality in immunocompromised patients. According to a recently published meta‐analysis, the odds of COVID‐19 disease are more than five times higher in HTX recipients compared to the general population and the mortality rate of the infected HTX individuals is also significantly higher (almost 30%).^8^ However, it is of note that the majority of these reports are from the era before vaccines became available. In the current single‐center analysis, the case fatality ratio of all HTX recipients including both vaccinated and unvaccinated patients was 6.5%.

Based on the literature, the percentage of HTX recipients achieving humoral immune response after two doses of SARS‐CoV‐2 vaccination ranges between 12% and 75% due to the high heterogeneity of the examined populations.^10^, ^11^, ^12^, ^13^, ^23^, ^24^ In our study, 62% of the HTX recipients became seropositive after two doses of the SARS‐CoV‐2 vaccine and 58% of the seronegative patients achieved seroconversion after a third, booster vaccination. Furthermore, a fourth vaccine dose resulted in seroconversion in a few more cases. Numerous studies confirmed that a third dose of vaccine increased antibody response against SARS‐CoV‐2 in solid organ transplant recipients.^11^, ^12^, ^13^, ^25^, ^26^, ^27^, ^28^, ^29^ In light of these findings, it became evident that booster vaccine doses would be essential in the prevention strategy against SARS‐CoV‐2. However, several recipients had insufficient immune response against SARS‐CoV‐2 indicating that alternative strategies may be needed to improve protection in some immunocompromised patients.^26^, ^27^, ^28^ For those solid organ transplant recipients who do not have sufficient antibody titers after three vaccines, the ESOT suggests the administration of additional booster vaccine doses or the consideration of pre‐exposure prophylaxis with monoclonal antibodies.^14^

In vaccinated HTX recipients, the weaker immune response was related to older age,^10^, ^23^, ^28^ male sex,^23^ vaccination within the first^23^ or second^28^ year after HTX and MMF or MPA immunosuppressant therapy.^10^, ^12^, ^23^, ^28^ Studies of solid organ transplant recipients showed that predictive factors for humoral response after vaccination were male sex, a longer period between transplantation and vaccination, living donor status, and liver transplantation. In contrast, older patients, recipients of a deceased donor organ, thoracic organ recipients, and those who received steroids or MMF/MPA‐based immunosuppressive regimen were less likely to develop anti‐SARS‐CoV‐2 antibodies.^13^, ^27^, ^29^, ^30^ A recent meta‐analysis verified that mRNA vaccines may cause a stronger humoral immune response compared to the inactivated vaccines in solid organ transplant recipients.^13^ In our study, significant predictors of seroconversion were mRNA vaccine, longer time between HTX and the vaccination, and male sex of the recipient. Lower seroconversion rate among females verified by both the aforementioned studies and our analysis, is an unexpected phenomenon. A recent translational study reported a significantly reduced uptake of cationic lipid nanoparticles by the natural killer cells of healthy female persons which may result in lower immune response to mRNA vaccines in females.^31^ However, the exact cause and relevance of this sex‐related difference warrants further research. Based on the aforementioned literature data, the current ESOT position statement recommends the SARS‐CoV‐2 vaccination of all transplant candidates before transplantation and the avoidment of vaccination in the first 3 months after transplantation.^14^ Two doses of SARS‐CoV‐2 vaccination was reported to be protective against symptomatic COVID‐19 in solid organ transplant recipients.^32^ Vaccination was also shown to be associated with fewer hospitalizations and deaths due to COVID‐19,^9^, ^15^ with no allograft dysfunction or clinically significant rejection.^9^ However, two doses of SARS‐CoV‐2 vaccination proved to be less protective against lethal outcome in solid organ transplant recipients.^15^ The prevalence of SARS‐CoV‐2 infection was 40% among our HTX recipients and one‐third of them experienced severe course of disease. Severe infection occurred in 37% of the unvaccinated or incompletely vaccinated group and in 27% of the completely vaccinated recipients throughout all pandemic waves caused by various—including less agressive—types of SARS‐CoV‐2. Most patients in the incompletely vaccinated group were infected with the original Wuhan strain or the Alpha variant before the availability of vaccination.

Recent publications have reported that older age of the recipient^15^, ^16^, ^29^ and certain immunosuppressive regimens^16^ predispose transplant recipients to severe SARS‐CoV‐2 infection. In the current study, seronegative status before infection has been proven to be a significant predictor of severe infection. Kuczaj et al. reported similar results: in their HTX population, none of the vaccinated and then infected patients (*n* = 6) had detectable antibodies after the vaccination and, on the other hand, none of the patients with detectable antibodies against SARS‐CoV‐2 spike protein contracted COVID‐19 disease (infection severity was not evaluated in this study).^33^ An analysis of thoracic and abdominal organ recipients showed a significantly lower rate of positive antibody response after vaccination in those patients who required hospitalization due to SARS‐CoV‐2 infection compared with nonhospitalized individuals.^29^ The ESOT also recommends to use the titer of anti‐spike IgG in specific cases to estimate the level of protection of transplant recipients against severe COVID‐19. The absence of any detectable antibody response indicates lack of effective protection against severe COVID‐19 and may identify patients in need for additional protective strategies.^14^

Our study has some limitations. During the COVID‐19 pandemic, asymptomatic patients were scheduled for follow‐up examinations sparsely, therefore, antibody measurements could not be performed at standard time intervals. Due to the same reason, tacrolimus and everolimus trough levels could not always be measured in close proximity to the time of vaccinations but at the nearest follow‐up visits. Second, the anti‐SARS‐CoV‐2 spike protein antibodies do not provide complete information about the immunogenicity of vaccination. Furthermore, seropositivity caused by previous asymptomatic infection could not be excluded in some cases. Antinucleocapsid test to detect previous SARS‐CoV‐2 infection was available at our Clinic until April 2021. Although the majority of patients were checked for antinucleocapsid antibodies around the time of vaccination (previous unknown infection was identified in three cases), but antinucleocapsid antibodies of those patients whom follow‐up visits were scheduled sparsely could not be measured.

## 5 CONCLUSIONS

Male sex, longer time since transplantation, and mRNA vaccine type promote seroconversion after SARS‐CoV‐2 vaccination in HTX recipients. Seropositivity—but not the sole number of vaccine doses—seems to be protective against severe SARS‐CoV‐2 infection. Thus, routine screening of anti‐SARS‐COV‐2 antibodies may help to identify patients at risk for severe COVID‐19 disease. These high‐risk patients may benefit from further booster vaccination or other prophylactic strategies including novel pre‐exposure antibody treatment.

## AUTHOR CONTRIBUTIONS

Szilvia Kugler took part in the clinical care and follow‐up of the patients, contributed in the organization of vaccinations, collected and summarized patient data, reviewed the relevant literature and was a major contributor in creating the database and writing the manuscript. Dorottya Katalin Vári also took part in collecting patient data, creating the database and searching relevant manuscripts. Dániel Sándor Veres prepared the statistical analyses. Ákos Király, Tímea Teszák, Nóra Parázs, Zoltán Tarjányi, Zsófia Drobni, and Zsófia Szakál‐Tóth participated in the clinical care and follow‐up of the patients, helped organizing vaccinations and antibody measurements and documented antibody data. Gyula Prinz was our infectious disease consultant who guided the vaccination of the patients. Pál Miheller took part in the conception and the design of the study. Béla Merkely took part in planning of the study and supervised the clinical care of the patients. Balázs Sax took a major part in the clinical care and follow‐up of the patients as well as in the organization of vaccinations and antibody measurements and was also in charge of supervising the manuscript. All authors read and approved the final manuscript.

## CONFLICT OF INTEREST STATEMENT

The authors declare no conflict of interest.

## ETHICS STATEMENT

We confirm that all methods were carried out in accordance with relevant guidelines and regulations. The study protocol has been approved by the Research Ethics Committee of the Medical Research Council of Hungary (No. IV/861‐1/2021/EKU). All patients signed informed consent.

## Data Availability

The data that support the findings of this study are available from the corresponding author upon reasonable request.
